# Tyrosine phosphatase *PTPN11/*SHP2 in solid tumors - bull’s eye for targeted therapy?

**DOI:** 10.3389/fimmu.2024.1340726

**Published:** 2024-03-05

**Authors:** Xun Chen, Steffen Johannes Keller, Philipp Hafner, Asma Y. Alrawashdeh, Thomas Yul Avery, Johana Norona, Jinxue Zhou, Dietrich Alexander Ruess

**Affiliations:** ^1^ Department of General and Visceral Surgery, Center for Surgery, Medical Center University of Freiburg, Freiburg, Germany; ^2^ Department of Hepatobiliary and Pancreatic Surgery, The Affiliated Tumor Hospital of Zhengzhou University, Zhengzhou, China; ^3^ German Cancer Consortium (DKTK), Partner Site Freiburg and German Cancer Research Center (DKFZ), Heidelberg, Germany

**Keywords:** SHP2, solid tumor, signaling pathway, tumor microenvironment, immune microenvironment, therapy resistance

## Abstract

Encoded by *PTPN11*, the Src-homology 2 domain-containing phosphatase 2 (SHP2) integrates signals from various membrane-bound receptors such as receptor tyrosine kinases (RTKs), cytokine and integrin receptors and thereby promotes cell survival and proliferation. Activating mutations in the *PTPN11* gene may trigger signaling pathways leading to the development of hematological malignancies, but are rarely found in solid tumors. Yet, aberrant SHP2 expression or activation has implications in the development, progression and metastasis of many solid tumor entities. SHP2 is involved in multiple signaling cascades, including the RAS-RAF-MEK-ERK-, PI3K-AKT-, JAK-STAT- and PD-L1/PD-1- pathways. Although not mutated, activation or functional requirement of SHP2 appears to play a relevant and context-dependent dichotomous role. This mostly tumor-promoting and infrequently tumor-suppressive role exists in many cancers such as gastrointestinal tumors, pancreatic, liver and lung cancer, gynecological entities, head and neck cancers, prostate cancer, glioblastoma and melanoma. Recent studies have identified SHP2 as a potential biomarker for the prognosis of some solid tumors. Based on promising preclinical work and the advent of orally available allosteric SHP2-inhibitors early clinical trials are currently investigating SHP2-directed approaches in various solid tumors, either as a single agent or in combination regimes. We here provide a brief overview of the molecular functions of SHP2 and collate current knowledge with regard to the significance of SHP2 expression and function in different solid tumor entities, including cells in their microenvironment, immune escape and therapy resistance. In the context of the present landscape of clinical trials with allosteric SHP2-inhibitors we discuss the multitude of opportunities but also limitations of a strategy targeting this non-receptor protein tyrosine phosphatase for treatment of solid tumors.

## Introduction

Increased throughput of homeostatic or reactivation of embryologic signaling pathways, which are extremely strictly regulated in physiologic conditions, is a very frequent pathophysiologic underpinning for oncogenesis, cancer cell survival and aggressiveness. Many different cellular processes and states can become disturbed as a result of mutations and increased or restarted expression of signaling node proteins or regulators. This may have an impact on the mechanism of the cell cycle and proliferative drive, modify systems governing cell survival and death, and potentially rewire metabolism, metaplasia and dedifferentiation, migration, invasiveness, and anoikis, all of which may facilitate metastasis (1). The interplay of several mediators enables the sophisticated realization of most of these signal transduction mechanisms.

Phosphorylation and dephosphorylation are frequent and significant posttranslational chemical changes of proteins that act as molecular switches in this setting. Here, phosphorylation “erasers,” or protein phosphatases, balance out phosphorylation “writers,” or protein kinases, which, in contrast to phosphatases, have long been important players in drug development. SHP2, a non-receptor protein encoded by the *PTPN11* gene, functions as a tyrosine phosphatase within the broader family of phosphatases. It is ubiquitously expressed throughout the body and regulates a variety of cellular biological processes, including invasion, metastasis, apoptosis, differentiation, migration, and proliferation of cells (2). It operates through a number of signaling cascades, involving pathways such as RAS-RAF-MEK-ERK, PI3K-AKT, JAK-STAT, NF-κB and PD-L1/PD-1. It may seem counterintuitive, but by dephosphorylating signal regulators, it generally enhances signaling rather than inhibits it.

It has been established that SHP2 is an oncogene that is frequently mutated in leukemia, particularly in youngsters, and in developmental abnormalities (such Noonan syndrome and LEOPARD syndrome, both RASopathies) (3, 4). In contrast, development and progression of different solid cancers has been linked to a relevant contribution of non-mutated, wild-type SHP2, according to several findings sparked by the continued examination of the function and contribution of SHP2 (5). Given the potential dual role in the initiation and advancement of distinct solid tumors, the outcomes are contingent on the specific engagement of SHP2 with primary signaling pathways—acting either as a tumor suppressor or as an oncogenic tumor promoter (6–8). For the latter, SHP2 has become a potential and attractive target, especially with the aim of interfering with tumor intrinsic RAS-RAF-MEK-ERK-pathway hyperactivation, even as a combination partner for the novel groundbreaking class of mutant-specific RAS-inhibitors (9).

Historically, promiscuous active sites and unfavorable catalytic pocket shapes have made targeting phosphatases extremely difficult. Consequently, the recent introduction of allosteric SHP2 inhibitors is regarded as a significant advancement, offering refined inhibition with enhanced selectivity (10).

Here, we briefly introduce the basic structure and molecular function of SHP2. More importantly, we also summarize the role of SHP2 in different solid tumor entities and their tumor microenvironment, immune escape, and therapy resistance. We further provide a context of ongoing clinical trials with allosteric SHP2 inhibitors and discuss opportunities and challenges of a strategy targeting this non-receptor protein tyrosine phosphatase for the therapy of solid tumors.

## Molecular structure of SHP2 and mechanism of activation

**Figure 1:**
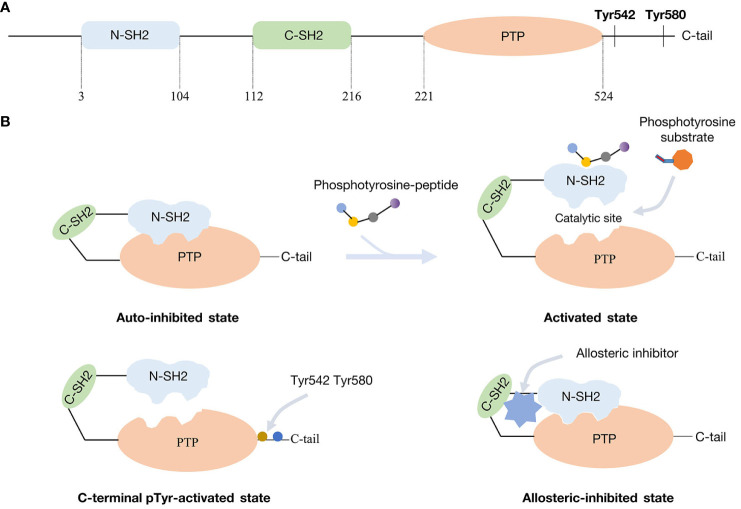
Molecular structure of SHP2 and mechanism of activation and inhibition: **(A)** SHP2 functional domains. **(B)** SHP2 in the inhibited and activated state from different manners.

SHP2 is a non-receptor phosphotyrosine phosphatase consisting of 593 amino acid residues (11). It carries two N-terminal Src-homology 2 domains, namely the N-SH2 and C-SH2 domains. Following these domains is the protein tyrosine phosphatase domain (PTP domain) and then a C-terminal tail that contains phosphorylatable tyrosine residues (**Figure 1A**) (12, 13). Through an intramolecular contact, the N-SH2 and PTP domains preserve the auto-inhibited conformation of SHP2, preventing the substrate from reaching the catalytic phosphatase site (14). Tyrosine-phosphorylated peptide motifs are used by upstream-activated proteins to recruit and bind the N-SH2 domain upon stimulation by various substances, including cytokines or growth factors. As an alternative or in addition, SHP2 can be phosphorylated at its C-terminal tyrosine residues, which may interact with the SH2 domain at the N-terminus. Self-inhibition is then relieved and the catalytic site for dephosphorylation activity is released (**Figure 1B**). The C-SH2 domain primarily serves the purpose of facilitating energy provision during the association involving the N-SH2 domain and SHP2 binding proteins. Given the possibility for adapter functions involving the two SH2 motifs and its phosphorylated C-terminal tyrosine residues, SHP2 may have responsibilities other than that of a phosphatase (15).

Growth factor receptors and scaffolding adapters, such as insulin receptor substrate (IRS), fibroblast growth factor receptor substrate (FRS), and GRB-associated binding protein (GAB) protein, are examples of binding partners for the SH2 domains of SHP2 (16). Quite frequently, hematological malignancies and Noonan/LEOPARD syndrome display *PTPN11* gene mutations. These activating mutations usually result in the loss of auto-inhibition. In leukemia patients, the most prevalent and active *PTPN11* variation is the SHP2^E76K^ mutation (17, 18). Yet, *PTPN11* mutations are uncommon in solid tumors - here, recruitment and activation of the wild-type form is of importance, at times accompanied by overexpression.

## Functional relevance of SHP2 in various signaling cascades and subcellular compartments

**Figure 2:**
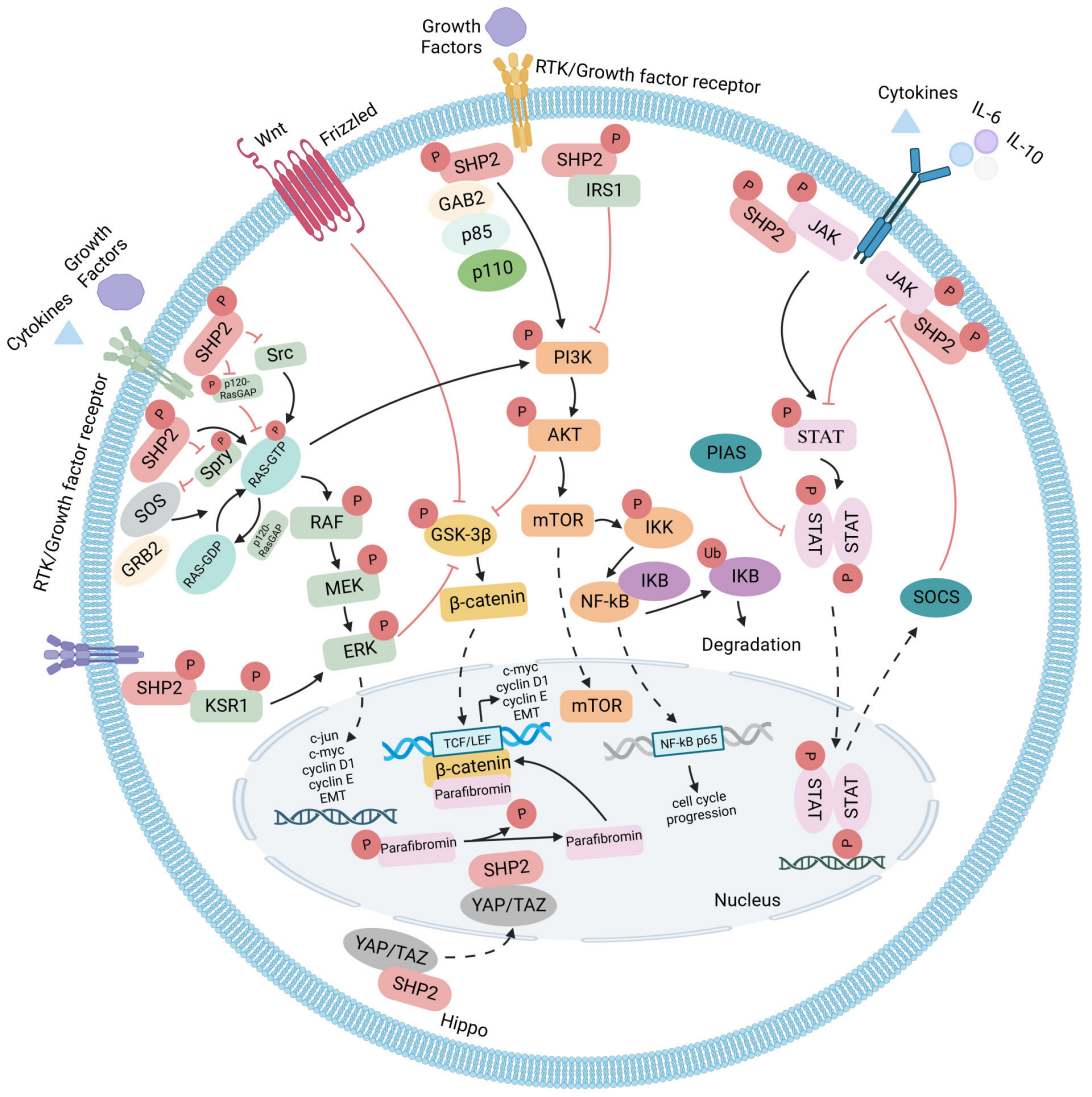
SHP2 signaling pathways: SHP2-dependent signaling in both developing and established cancer cells is illustrated, with arrows denoting positive regulators and T-bars indicating negative regulators. Functioning as a convergence node, SHP2 exhibits diverse roles in cell survival-related signaling pathways. It not only positively influences the RAS-RAF-MEK-ERK pathway but also modulates the PI3K-AKT and JAK-STAT pathways, either enhancing or antagonizing based on extracellular stimuli and the cellular environment. Additionally, SHP2 is involved in Wnt/β-catenin and NF-κB pathways. Nuclear localization of SHP2 has been observed in various contexts, and in the Hippo signaling pathway, YAP and TAZ act as the rheostat for nuclear SHP2 function.

SHP2 functions as a cytoplasmic downstream signaling molecule, establishing direct interactions with diverse membrane-bound receptors. Additionally, it associates with various signal transduction mediators, such as phosphoinositide 3-kinase (PI3K), Janus kinase 2 (JAK2), and growth factor receptor binding protein 2 (GRB2). As a result, SHP2 takes part in several signal cascades, including those involving RAS-RAF-MEK-ERK, PI3K-AKT, JAK-STAT, but also Wnt/β-catenin and NF-κB pathways (summarized in **Figure 2**). Furthermore, SHP2 has been ascribed playing roles in the mitochondria and in the nucleus. Through this array of functions, SHP2 regulates cell differentiation, survival, as well as proliferation, and consequently, influences tumor development and progression (19).

### RAS-RAF-MEK-ERK

The activation of the RAS/ERK pathway is incomplete without the involvement of SHP2 (20). When stimulated by growth factors (such as epidermal growth factor [EGF], platelet-derived growth factors [PDGFs]), active receptor tyrosine kinases (RTKs, including epidermal growth factor receptor [EGFR], epidermal growth factor receptor type 2 [HER2], platelet-derived and fibroblast growth factor receptors [PDGFRs]) or active cytokine-receptors (such as interleukin 3 [IL-3], interleukin 6 [IL-6], and granulocyte macrophage-colony stimulating factor [GM-CSF]) attract SHP2 by exposing phosphorylated tyrosine residues. The C-terminal tyrosine residues of SHP2 may then be also phosphorylated as a mechanism of activation. However, this phosphorylation is not necessary for SHP2’s autoinhibited conformation to be relieved and for it to be ready for phosphatase action. In order to activate downstream effectors, SHP2 will ultimately dephosphorylate downstream signaling molecules. SHP2, however, may also function as a relatively low-dependence adapter protein, bringing proteins in close proximity for interaction, based on its SH2-domain characteristics.

For full activation of the RAS pathway, both of these molecular roles are important: SHP2 serves as an adapter protein by recruiting phosphotyrosine binding substrates like GRB2, insulin receptor substrate 1 (IRS1), fibroblast growth factor receptor substrate 2 (FRS2), Src homology, and collagen homology (Shc) to the membrane receptor tyrosine kinases in response to extracellular stimuli. GAB1/2, or GRB2-associated-binding proteins 1/2, are significant SHP2 binding partners. They have two SHP2 binding sites. These sites are first autophosphorylated to create a double-phosphorylated tyrosine activation motif during GAB1/2 activation. Subsequently, this motif binds to the two SH2 domains of SHP2, leading to the release and activation of the closed conformation within the SHP2 molecule. The Son of Sevenless (SOS) proteins aggregate at the cell membrane as a result of GRB2 binding to phosphorylated growth factor receptors or SHP2 binding to GRB2 via GAB1/2. The membrane-binding protein RAS can be catalyzed by SOS, a guanine nucleotide exchange factor (GEF), to transition from its inactive RAS-GDP state to the active RAS-GTP state. RAS-GTP then acts as a bridge to downstream signaling systems, triggering the activation of the RAF-1 serine/threonine kinase and amplifying the kinase cascade via MEK1/2 kinase, which in turn activates ERK1/2. Following activation, ERK1/2 regulates gene transcription by acting directly on cytoplasmic target molecules or translocates to the nucleus, which in the end promotes cell proliferation or differentiation (21, 22).

In order to transfer cascade activation signals, SHP2 may also indirectly bind to Sprouty (Spry), p120 RAS GTPase activating protein (p120 RAS GAP), and Src family kinase (SFK) in the RAS-RAF-MEK-ERK pathway (23–26). These scaffold proteins with their conserved tyrosine residues not only operate as adaptor proteins but also have the ability to quickly become dephosphorylated through interaction with SHP2, which sets off a series of stimuli signals. For example, in an inactivation/baseline state, mouse Sprouty2 (mSpry2) can form complexes with SHP2, GAP1 and GRB2, distant from the plasma membrane. Spry’s “trap effect” is caused by the binding between SHP2 and Spry2, which also keeps Spry dephosphorylated. Upon stimulation with e.g. fibroblast growth factor 10 (FGF10), phosphorylated Spry2 disengages from diverse complexes and relocates to the cell membrane. Subsequently, the adaptor protein FRS2 recruits the GRB2/SOS complex, facilitating the FGF10-induced MAPK cascade signaling (24, 27).

Via a positive signaling route, SHP2 can also facilitate EGF-induced RAS activation by dephosphorylating p120 RAS GAP (20). Thus, the half-life of RAS binding to GTP is significantly extended, prolonging RAS activation, since RAS-GAP, in this state, is unable to translocate to the plasma membrane for interaction with RAS (22).

Moreover, SRC and SHP2 dynamically regulate a tyrosine phosphorylation of KRAS at Tyr32 and Tyr64, contributing to the molecular switching role of RAS and influencing GEF-mediated nucleotide exchange (28, 29).

In addition, a recent study suggests that SHP2 interacts with the scaffold protein kinase suppressor of RAS1 (KSR1). This interaction results in the detachment of KSR1 from activated SHP2, followed by anchoring to the plasma membrane through the dephosphorylation of Tyr25, facilitating ERK signal transduction (30).

While the mechanistic roles of SHP2 in RAS-activation are manifold and context-dependent, a number of biochemical and genetic studies have now accumulated evidence that SHP2 is an important upstream regulator of even oncogenic mutant RAS isoforms (31, 32). Genetic knockout or pharmacological inhibition of SHP2 has the potential to inhibit full RAS/MAPK signaling thereby limiting progression of a range of tumors dependent on activated RTKs but also those with mutant RAS oncoproteins (6, 8, 33).

### PI3K-AKT

Activation of SHP2 downstream of growth factor receptors also governs the growth and survival signaling pathway of PI3K-AKT kinase (34, 35). PI3Ks, a family of lipid kinases, are essential for regulating diverse cellular processes such as development, proliferation, apoptosis, migration, and metabolism. Several upstream factors, such as RTKs or G-protein-coupled receptors, which cause PI3K recruitment to the membrane, can activate PI3K (36). As previously noted, receptor dimerization and autophosphorylation of tyrosine residues on receptor tyrosine kinases (RTKs) occur upon ligand binding. This process is followed by interactions with molecules that possess an SH2 domain (37). PI3Ks can be activated in three distinct manners (1): The activation of the catalytic subunit p110 of PI3Ks is triggered by the interaction of the regulatory subunit p85 of PI3Ks with phosphorylated motifs within RTKs (38) (2); It preferentially attaches to the phosphorylation motifs of RTKs as well as the scaffold protein GAB1/2 can subsequently bind to p85 (39) (3); RAS binding: GRB2 binds and activates SOS, thereby instigating RAS activation, which activates p110 autonomously from p85 (40).

PI3K regulatory subunit SH2 domain binding affinity can be directed toward phosphotyrosine residues of RTK or adaptor proteins, including IRS1, facilitating activation. This activation can occur both in an RTK-dependent and RAS-independent manner (41). PI3K’s upstream molecule IRS1 is in charge of attaching to and continuously activating PI3K, which in turn recruits downstream AKT (42, 43). Partially because of the connection between IRS-1 and SHP2, some PI3K binding motifs get dephosphorylated. Subsequently, protein synthesis is diminished, and the PI3K cascade signaling pathway is inhibited. Complete activation of AKT occurs through phosphorylation at the hydrophobic motif S473 in the carboxy terminus via the mammalian rapamycin complex 2 target (mTORC2) (44). After AKT is phosphorylated, several downstream targets crucial for controlling cell survival and death are also phosphorylated. By phosphorylating and deactivating a 40 kDa proline-rich AKT substrate (PRAS40) and tuberculosis 2 (TSC2), phosphorylated AKT stimulates the mammalian rapamycin target (mTOR) (45). Moreover, AKT prevents the phosphorylation of glycogen synthase kinase 3 (GSK-3), thereby stimulating the synthesis of cell cycle regulatory molecules like c-myc, cyclin D1, and cyclin E (46). Remarkably, AKT has the potential to activate the IκB kinase (IKK) and the NF-κB signaling pathway. To summarize, the IKK/NF-κB signaling pathway is implicated in multiple phases of cancer development and progression (47).

PI3K’s stimulation in reaction to growth factors via RAS involves a two-stage process. Phosphorylation of RTKs, which are occasionally adapter proteins, is the initial stage. Then RAS is activated in the second phase of the process. RAS-GTP interacts with PI3K p110α. Their direct contact increases the activity of p110α. This can be done by bringing substrate binding sites into a new conformation, boosting their catalytic activity, or facilitating tighter connections with the plasma membrane. RAS is primarily employed to stabilize PI3K on the plasma membrane, which is triggered by conformational changes in RTK, for RTK-induced mammalian PI3K activation (48). The activation of p110α results from the disruption of the inhibitory contact between SH2 and the catalytic subunit. This occurs when the ligand binds to the RTK, connecting the phosphorylated tyrosine motif in the receptor to the SH2 domain of p85 (49). The separation of p110 and p85 reduces the inhibition of p85 for Ras-mediated PI3K activation, and p85’s SH2 domain is essential (50). The PI3K catalytic subunit p110 is directly bound by the active RAS at amino acids that are distinct from those needed for p85 binding.

Determining whether a given stimulus activates PI3K through RAS-dependent, p85-independent, or RAS-independent processes is often challenging, as many stimuli activating PI3K also trigger activation of both p85 and RAS (51). As a result, SHP2 plays a variety of intricate roles in these processes, which can be redundant, stimulating, or inhibiting, depending on the situation.

### β-catenin

Numerous pieces of evidence indicate that β-catenin stability is also influenced by tyrosine kinase activation. For instance, several tyrosine residues in EGFR, c-MET, and RON can be directly phosphorylated by active RTK, which increases transcriptional activity and protein stability for β-catenin. Furthermore, it has been reported that activated downstream signaling molecules, including AKT and ERK1/2, phosphorylate inhibitory Ser9 residues, leading to the inactivation of glycogen synthase kinase 3β (GSK-3β) and consequently stabilizing β-catenin (52). SHP2 serves as an upstream regulator in this signaling pathway. Given that RTK activation is the primary mechanism for initiating signal transduction via the RAS-RAF-MEK-ERK and PI3K-AKT pathways, SHP2 may mediate RTK induction, influencing the stability of β-catenin. In certain instances, SHP2-dependent β-catenin stability may be essential for tumor development, cell proliferation, transformation, and metastasis (53). Similarly, the β-catenin signaling pathway is implicated in the induction of epithelial-mesenchymal transition (EMT), possibly influenced by SHP2 (54).

It is noteworthy that parafibromin, a tumor inhibitor, recruits SUV39H1 histone methyltransferase to block cyclin D1 and c-myc (55). Nevertheless, β-catenin proteins that bind to parafibromin can also operate in the reverse way, triggering the pro-mitotic/carcinogenic Wnt signaling pathway (56). Only after tyrosine dephosphorylation by SHP2, parafibromin acquires stable binding β-catenin abilities. The parafibromin/β-catenin interaction then leads to an augmentation in the expression of Wnt target genes, including c-myc and cyclin D1 (57).

### JAK-STAT

In governing how cells respond to extracellular cytokines, the JAK-STAT pathway is of vital importance. Seven STAT members (STAT1, STAT2, STAT3, STAT4, STAT5a, STAT5b, as well as STAT6) and four JAK members (JAK1, JAK2, JAK3, and TYK2) have been found in mammalian cells. The several subtypes point to the JAK-STAT pathway’s functional complexity. JAKs belong to the non-receptor tyrosine kinase family. STAT proteins engage in the transcription and expression of a multitude of target genes within the nucleus concurrently. These mechanisms are essential for various cellular processes, encompassing immunological control, differentiation, cell survival, and inflammation. The primary downstream effector of cytokines, such as type I/II interferon and many interleukins, including IL-6 and IL-10, is the JAK-STAT pathway (58, 59). The current framework of the JAK-STAT signaling cascade posits that receptor oligomerization occurs when cytokines bind to their respective cell surface receptors; this oligomerization then activates JAK tyrosine kinases that are correlated with the receptor. By phosphorylating the tyrosine residues of the receptor, activated JAK creates binding sites for STAT recruitment and phosphorylation (60). Following dimerization, phosphorylated STATs dissociate from the receptor and translocate to the nucleus, initiating the transcription of genes (61).

Multiple strategies can be employed to modulate JAK-STAT signaling at different stages. The suppressor of cytokine signaling (SOCS) protein and the protein inhibitor of the activated STAT (PIAS) family, as well as diverse protein tyrosine phosphatases (PTPs) are important regulatory elements. SHP2 may either strengthen or weaken the JAK-STAT pathway according to substrate specificity, much like the two-edged sword in the PI3K-AKT signaling pathway (62).

On the one hand, SHP2’s dephosphorylation activity can influence downstream transcriptional regulation and STAT activation favorably. Phosphorylation controls the activity of JAK2 kinase in a site-dependent fashion. The IL3-driven JAK2-STAT5 signal is impaired in SHP2 mutant cells, yet wild-type SHP2 has the potential to reactivate this signal. The lack of SHP2 also strongly suppresses the activity of STAT5 (63, 64). SHP2 inactivation results in a decrease in both JAK2 activity and STAT5 phosphorylation (65). The JAK-STAT signal is inhibited when the Tyr1017 phosphorylation site of JAK combines with SOCS to generate a complex that stops JAK from binding to STAT. Through JAK’s tyrosine phosphorylation site dephosphorylation, SHP2 can stop JAK from attaching to SOCS and restart the STAT signaling cascade.

SHP2, however, also has the ability to inhibit the JAK-STAT pathway (7, 66). For example, SHP2 is drawn to the phosphorylated tyrosine residue pTyr759 of the gp130 cytokine receptor subunit, where it consequently can dephosphorylate STAT3, blocking JAK-STAT3 signaling, and IL-6-induced gene activation (67). Along this line, Ohtani et al. (62) showed that gp130-induced extension of STAT3 activation was present in animals devoid of SHP2 signaling, suggesting a negative function for SHP2 in JAK-STAT signaling.

### NF-κB

Nuclear factor kappa-light-chain-enhancer of activated B cells (NF-κB) is a crucial transcription factor involved in transmitting signals from interleukin-1 (IL-1) and tumor necrosis factor (TNF) stimulation in various physiological and pathological processes (68, 69). The nuclear factor binding of light peptide gene enhancers generates inactive complexes and is in an inhibitory state in the basic state of NF-κB and B-cells inhibitor (IκB) (70). Phosphorylation of inhibitory-κB kinases (IKKs) by numerous kinases, such as AKT, mTOR, as well as mitogen-activated protein kinase (MAPK), lead to the activation of NF-κB (71–73). The transcriptional activity of NF-κB can be regulated by the RAS oncoprotein through its targeting of the transactivation domain of the NF-κB p65 subunit. SHP2 has been shown to be an essential component for the regulation of NF-κB *in vivo*. In the context of RAS mutant NSCLC, the activation of the NF-kB pathway upstream of IκB was induced by SHP2 inhibition. Within this particular framework, the inhibition of the SHP2-RAS-ERK pathway results in an upregulation of CXCR2 ligands that is dependent on NF-κB. This, in turn, results in the recruitment of S100A8^high^ granulocytic myeloid-derived suppressor cells (MDSCs), which exert suppressive effects on T cells (74).

### Mitochondria

It has been demonstrated that SHP2 is present in mitochondria, which are multipurpose organelles engaged in a number of biological functions such as energy synthesis, intermediate metabolism, cell apoptosis, and preservation of cytoplasmic calcium homeostasis (75, 76). However, most research efforts have concentrated on non-solid tumors and non-neoplastic disorders. For instance, Zang et al. (77) showed that alterations in the mitochondrial location of tyrosine kinase Src and tyrosine phosphatase SHP2 are responsible for sepsis-induced cardiac mitochondrial dysfunction. Protein tyrosine phosphatase *PTPN11* tumor-related activation mutations increase mitochondrial metabolism, which causes oxidative stress and aging (78). Then, Guo et al. (79) found that SHP2 controls the stimulation of the NLR family pyridine-containing domain 3 (NLRP3) inflammasome negatively by affecting the homeostasis of the mitochondria, which is reliant on adenine nucleotide transferase 1 (ANT1). Considering the significance of mitochondria, SHP2’s influence on mitochondrial activity may play a part in mediating its involvement in solid tumors. However, currently there is little information available, therefore more research is necessary.

### Nucleus

A small portion of SHP2 has been shown to be present in the nucleus, and there is evidence that SHP2 contributes to certain nuclear processes (80). SHP2 has the ability to trigger DNA damage-induced cell death and apoptosis, both of which are p53-independent processes. Furthermore, DNA damage-induced translocation of cell division cycle 25C (Cdc25C) from the nucleus to the cytoplasm requires SHP2 (81). Furthermore, it has been demonstrated that nuclear SHP2 controls STAT transcription factors. Through the nuclear interaction of SHP2 with telomerase reverse transcriptase (TERT), TERT is prevented from being exported from the nucleus, which decreases apoptosis sensitivity and prevents accelerated senescence of cells (80, 82). The aforementioned SHP2-mediated parafibromin dephosphorylation has also been reported to occur in the nucleus. The β-chain protein binds to dephosphorylated parafibromin, which is a part of the elongation factors PAF1 complex (PAF). When Wnt target genes are activated, the resultant parafibromin/β-catenin protein complex functions as a co-activator of T cell-specific transcription factor/lymphoid enhancer factor (TCF/LEF) (57).

Yes-associated protein (YAP) is linked to another nuclear function of SHP2. The nuclear accumulation of SHP2 is considerably facilitated by the interaction between dephosphorylated YAP and the transcription coactivator with PDZ binding motif (TAZ). SHP2 is kept in the cytoplasm by YAP/TAZ phosphorylation via Hippo signaling, which inhibits its nuclear function (83). The prognostic significance of SHP2 may be attributed to its nuclear localization and interaction with nuclear YAP1. This interaction activates the Wnt/ß-catenin signaling pathway, resulting in elevated expression of cyclin D1 and the c-myc (84).

In conclusion, SHP2 has been found in the nucleus across various contexts, including solid tumor types. This localization holds the potential to influence gene expression and may be crucial for cell proliferation, survival, and other facets of carcinogenesis. Future research should focus further on the function of nuclear SHP2 in epigenetic alterations, tumor growth, gene regulation, and therapeutic targets.

## The role of SHP2 in different solid tumor entities

### Lung cancer

First of all, mutations that activate *PTPN11* can actually be detected in lung cancer. E76V, E76K, V45L, and N58S were reported before (85–87). A variety of 23 distinct *PTPN11* mutations were discovered when Richards et al. (87) recently analyzed *PTPN11* mutations in 37 *PTPN11*-mutant non-small-cell lung cancer (NSCLC). The mutations were listed from high to low frequency: E76A, A72D, S502L, G503V, N58S, G60D, E76K, M82V, E225, E313, E412G, Y418, D425Y, H426R, V428M, A452S, G483D, Q500E, G503R, M504I, Y521F, D551N, D556N. Remarkably, exons account for 75% (26/37) of the NSCLC *PTPN11* mutations, suggesting that the majority of *PTPN11* mutations are functionally relevant in NSCLC. In a genetically modified mouse model, the *PTPN11* E76K mutation is sufficient to cause NSCLC (86).

However, SHP2 appears to hold broader significance. In lung cancer tissue, wild-type SHP2 expression is markedly elevated compared to surrounding normal lung tissue, establishing a robust correlation between high SHP2 expression and lymph node metastasis. The overexpression of SHP2 might promote invasion and metastasis in NSCLC through processes like angiogenesis and lymphatic diffusion (88, 89). SHP2 knockdown lowers ERK phosphorylation and increases cell susceptibility to the EGFR inhibitor gefitinib in wild-type NSCLC cells expressing EGFR (90). As demonstrated by Dardaei et al. (91), where SHP2 inhibition prevents the reactivation of RAS and ERK1/2 following treatment with intermediate degenerative lymphoma kinase (ALK) inhibitors in NSCLC, the synergistic impact of combined RTK and SHP2 inhibitors appears to be a more general effect.

Furthermore, as shown by Mainardi et al. (8), knockout of *PTPN11* in NSCLC cells has little effect on cell proliferation but increased sensitivity to MEK inhibition. In KRAS mutant NSCLC, blocking SHP2 can cause a senescence response, whereas inhibiting MEK can aggravate this process. More thorough analyses of SHP2 inhibitor treatment in KRAS- and EGFR-mutant NSCLC models revealed tumor-intrinsic CCL5/CXCL10 secretion. This secretion resulted in the depletion of alveolar and M2-like macrophages while inducing and promoting infiltration of B and T lymphocytes. This was not surprising, given the crosstalk of senescent cells with their immune environment. Furthermore, by simultaneously blocking the CXCR1/2-dependent import of immunosuppressive MDSCs, combination inhibition of SHP2 and CXCR1/2 may enhance antitumor T cell responses in NSCLC (74). Additionally, elevated SHP2 expression correlates with enhanced survival in advanced KRAS mutant NSCLC and serves as a predictor for the efficacy of PD-1/PD-L1 therapies (92).

In conclusion, SHP2 appears to be a promising therapeutic target in combination treatment methods for NSCLC since it plays a critical positive regulatory function in the incidence, metastasis, immune evasion, and drug resistance of lung cancer, regardless of individual RTK/RAS mutational status.

### Pancreatic ductal adenocarcinoma

About 90% of PDAC tumors bear a KRAS mutation, which might encourage precancerous lesions associated with pancreatic cancer to progressively progress into invasive malignancy (93). Zheng et al. (94) used immunohistochemical techniques in order to assess the expression of SHP2 protein in 79 PDAC samples. Their results revealed that the proportion of PDAC tissues with high SHP2 expression was considerably greater (55.7 percent) than that of adjacent noncancerous tissues (10.1 percent). Additionally, it was observed that individuals with high expression of SHP2 had a shorter overall survival time in comparison to those with low expression. Studies have indicated a possible correlation between the onset and progression of PDAC and elevated expression of SHP2, indicating SHP2’s potential in both prognostic and therapeutic capacities. According to a different study, SHP2 expression is detectable for the duration of the PDAC tumor formation process. Furthermore, in a genetically altered mouse model, the generation of mutant KRAS-dependent PDAC was virtually entirely inhibited and survival time was markedly increased with deletion of *PTPN11*.

In established PDAC tumors, loss of SHP2 (or inhibition) sensitized to MEK inhibition and synergistically reduced tumor growth (6). Fedele et al. (95) discovered that SHP2 inhibition reduced RTK feedback signals and enhanced the action of KRAS^G12C^ inhibitors in PDAC. Furthermore, the SHP2-PDHA1-ROS axis is crucial for the upkeep of adipocytes and may regulate cytokine production as well as the proliferation of pancreatic cancer cells (96). Consequently, the majority of recent research points to a favorable association between SHP2 and the onset and progression of PDAC, indicating that SHP2 might be a desirable target in treatment combinations for this predominantly KRAS-mutated entity.

### Hepatocellular carcinoma and cholangiocarcinoma

According to a preliminary study, the deficit in SHP2 specific for liver cells drives inflammatory signaling through the STAT3 pathway, resulting in tumor formation and regenerative proliferation in aged mice. Hepatocellular carcinoma (HCC) produced by diethylene nitrite (DEN) is considerably more likely to develop when SHP2 is absent, but this is eliminated when SHP2 and STAT3 are both absent at the same time in liver cells (7). According to this study, *PTPN11*/SHP2 may have a tumor-suppressive function in HCC that is driven by inflammation.

In addition, Chen et al. (97) have shown that Myc-driven HCC is dramatically aggravated in mice with hepatocyte-specific *Ptpn11*/Shp2 deletion.

Subsequent research revealed that liver cancer spheroids rich in patient-derived CSC as well as cancer stem cells (CSCs) selected for epithelial cell adhesion molecule (EpCAM) or differentiation cluster 133 (CD133) showed a substantial increase in SHP2. By encouraging the dedifferentiation of liver cancer cells and boosting the self-renewal of liver stem cells, upregulated SHP2 facilitates the proliferation of hepatic CSCs (98). According to this study, β-catenin signaling amplifies SHP2, which in turn stimulates the proliferation of hepatic CSC and the dedifferentiation of HCC cells. This information could be utilized to anticipate how a patient will react to chemotherapy. Furthermore, a recent study revealed that patient-derived xenografts and developed sorafenib-resistant cell lines have much higher levels of SHP2. By obstructing feedback pathways, the SHP2 inhibitor SHP099 may eradicate sorafenib resistance in organoid cultures and HCC cell lines (99). SHP2 inhibition decreases adaptive resistance to sorafenib by preventing the reactivation of the MEK/ERK and AKT signaling pathways induced by RTK, according to the findings of this study. In HCC, mTOR and SHP2 inhibition have been shown to have a similar synergistic impact (100).

Growth arrest and DNA damage 45G (GADD45G) were also reported to be generally downregulated in human and mouse HCC (101) and oncogene-transformed mouse liver cells. GADD45G ectopic expression significantly accelerates the aging process of HCC and suppresses tumor development *in vivo*. In human hepatocellular carcinoma cell lines, GADD45G expression strongly suppresses constitutive phosphorylation of STAT3 (Tyr^705^). Since GADD45G-induced STAT3 dephosphorylation was successfully reduced and senescence induction was greatly suppressed, it suggests that this action is reliant on SHP2.

Cholangiocarcinoma has been reported to have similar processes. Additionally, in this case, Sorafenib-induced dephosphorylation of Tyr^705^ STAT3 is mediated by SHP2, and in cholangiocarcinoma cells, Sorafenib-induced dephosphorylation of Tyr705 p-STAT3 is inhibited by knocking down SHP2. This increases tumor cell death (102).

Conversely, SHP2 has been demonstrated to function as a tumor suppressor by preventing YAP-mediated cholangiocarcinoma development (103).

Then, and this is crucial, SHP2 inhibitors can boost the antitumor innate immune system in liver cancer driven by RTKs by upregulating interferon-β Secretion, downregulating inflammatory cytokines, and inhibiting the chemokine receptor 5 signaling axis (104).

Consequently, SHP2 has two distinct functions in primary liver cancer: promoting and/or suppressing tumor growth in hepatocellular carcinoma and cholangiocarcinoma.

As a consequence, therapeutic approaches targeting SHP2 in liver cancer would need to be tailored in a context and time-dependent fashion.

### Gastric cancer and esophageal cancer

According to related research, the expression rate of SHP2 in gastric cancerous tissues is notably greater than in healthy stomach mucosa (105). All identified cancer cell lines express SHP2 and certain diffuse gastric cancer (DGC) cell lines with MET or fibroblast growth factor receptor 2 (FGFR2) gene amplification exhibit preferential tyrosine phosphorylation. Treatment with MET or FGFR inhibitors or their deletion significantly reduces SHP2 tyrosine phosphorylation. In a mouse xenograft model, pharmacological inhibition or suppression of SHP2 effectively inhibits the migratory, invasive, and peritoneal dissemination of DGC cells that are dependent on MET (106).

Significant overexpression of KRAS protein was seen in the KRAS amplified gastroesophageal adenocarcinoma (GEAC) model. While being resistant to MAPK inhibition, mechanistically the model was able to respond adaptively by quickly boosting KRAS GTP levels. The adaptation process was decreased by the suppression of guanine exchange factors SOS1 and SOS2 or protein tyrosine phosphatase SHP2, and the model became more sensitive to MEK inhibition when these components were targeted, as shown by Wong et al. (107).

With regards to KRAS mutant gastric cancer, Zheng et al. (30) recently discovered that, via suppressing KSR1 activity, SHP2 inhibition mitigates adaptive resistance to MEK inhibitors.

A different role for SHP2 in human esophageal squamous cell carcinoma (ESCC) was discovered by Xu et al. (67). SHP2 expression appeared to be frequently downregulated in ESCC tissues. Additionally, patients exhibiting low SHP2 expression experienced poorer overall survival (OS) compared to those with higher SHP2 expression levels. SHP2’s functional implications for blocking inflammatory STAT3-signaling appear to be predominant in this context. By dephosphorylating STAT3, SHP2 appears to impair growth and progression of ESCC.

In conclusion, SHP2 is recognized for its oncogenic function in the genesis and advancement of GEAC, but not in ESCC. Furthermore, it could be a clinical treatment target for GEAC and ESCC as well as a prognostic marker.

### Colorectal cancer

Early research revealed that colorectal adenomas and stage 1 tumors had much higher levels of SHP2 expression than did later advanced cancers (108). SHP2 may have opposing effects in the large intestine cancer: depending on the inflammatory milieu, it may either stimulate or prevent tumor growth. The proliferation, invasion, and tumor properties of intestinal epithelial cells (IECs) derived from human colorectal cancer cells (CRC) and carcinogenic KRAS are inhibited by SHP2 silencing. SHP2 was required in IEC and CRC cells harboring oncogenic KRAS for full activation of MEK/ERK signaling. In addition, inhibition or knockout of *PTPN11* confers sensitivity to BRAF inhibition in BRAF mutant colon cancer (109).

Conversely, Huang et al. (110) were able to show that SHP2 inhibits the proliferation and migration of CRC cells by dephosphorylating STAT3. As per the findings, SHP2 inhibition and knockdown boost cellular signaling in HCT-116 colorectal cancer cells, hence promoting their proliferation both *in vitro* as well as *in vivo* (111).

The following two examples further highlight the difficulty in identifying and discerning tumor-promoting and tumor-suppressing functions, even though targeting SHP2 in frank colon cancer may be beneficial: first, SHP2 appeared to worsen the activation of the interferon gene stimulatory factor (STING) pathway by limiting the DNA repair mediated by poly ADP-ribose-polymerase 1 (PARP1) in colorectal cancer cells, indicating that SHP2 agonist lovastatin combined with chemotherapy is a viable treatment option for colorectal cancer (112). Second, and in contrast, cetuximab and EGFR-targeted therapy has long been an approved treatment protocol for RAS wild-type, metastatic colorectal cancer, and SHP2 inhibitors reliant on phospholipase C gamma 1 (PLCg1) exhibit anti-tumor effects in cancer cells resistant to cetuximab (113).

As a consequence, SHP2 inhibition approaches in colorectal cancer, although promising, may have mixed effects and need to be closely monitored with respect to their intended beneficial and potentially detrimental components.

### Oral squamous cell and laryngeal cancer

Research has also been done on the function of SHP2 in the progression of oral cancer, particularly oral squamous cell carcinoma (OSCC) (114). SHP2 protein expression is significantly increased in OSCC tissues compared to normal tissues. This overexpression is correlated to clinical staging and lymph node metastasis in advanced tumors. By controlling the expression of proteins linked to apoptosis, inhibiting SHP2 expression *in vitro* can cause cell death and reduce OSCC cell viability and invasion (115). Further, research has demonstrated that SHP2 promotes the metastasis of oral cancer cells. This suggests that the SHP2-ERK1/2-Snail/Twist1 pathway may play a significant role in the invasion and metastasis of oral cancer (116). In addition, deletion of SHP2 also exhibits significantly reduced metastatic capacity in OSCC.

In conclusion, SHP2 could be an oncogenic gene that encourages OSCC metastases. It may be investigated further as a biomarker for estimating how OSCC will develop, and focusing on SHP2 has been proposed as a novel approach for OSCC clinical management in the future.

It was discovered that SHP2 contributes to proliferative signals for laryngeal cancer cells as well, a process which is, again, mainly mediated via the MAPK pathway (117). Thus, SHP2 may contribute to the carcinogenicity of laryngeal cancer through involvement in the RAS/RAF/MEK/ERK signaling pathway and could be a useful target for therapy.

### Thyroid cancer

After examining 65 individuals with thyroid cancer (TC), Hu et al. (118) discovered that the samples and cell lines of thyroid cancer patients had elevated levels of SHP2. In the meanwhile, the findings demonstrated a strong association between high SHP2 expression and lymph node metastases, high TNM staging, and tumor differentiation.

On the other hand, TC patients with SHP2 overexpression had a reduced probability of passing away from the illness than did TC patients with low SHP2 expression, according to findings by Cao et al. (119). SHP2 expression did not, however, function as an independent predictive factor in multivariate analysis.

SHP2 blockade can overcome MEK inhibitor resistance, consistent with the findings of studies on other tumor types and leads to TC growth suppression that is persistent, particularly in differentiated thyroid carcinoma (DTC) cells (120). On the basis of these concepts, Ma et al. (121) recently showed that vemurafenib-induced cancer sensitivity was enhanced by inhibiting SHP2, which also reversed the reactivation of the MAPK/ERK signaling cascade brought on by RTK activation.

It’s interesting to note that, because SHP2 is necessary to propagate the PD-1 signal in this context, blocking the PD-1 circuit in TC might directly harm the proliferation of TC cells expressing PD-1. This inhibition would not only suppress the RAS/MAPK signaling pathway but also may restore anti-cancer immunity (122).

When considered together and in light of recent findings, SHP2 seems to be involved in the carcinogenesis of thyroid cancer, and blocking it might be a useful adjunctive therapeutic approach.

### Breast cancer

Research indicates that SHP2 is one of the essential signal transduction molecules that activate the RAS/ERK pathway in breast cancer as well and that this pathway plays a significant role in the occurrence and progression of breast cancer (123). For the first time, Zhou and Agazie (124) hypothesized that breast cancer cells had elevated levels of SHP2. They found that inhibiting SHP2 resulted in reduced EGF-induced RAS/ERK and PI3K/AKT signaling pathways, and brought back the typical phenotype of differentiated breast epithelium. The inhibition of SHP2 resulted in the downregulation of the mesenchymal markers vimentin and fibronectin and the upregulation of the epithelial marker E-cadherin. These findings suggest that SHP2 actively regulates mitosis, cell survival signaling, and EMT, hence promoting the phenotype of cancer cells. Therefore, it is not surprising, that SHP2 is overexpressed in invasive ductal carcinoma (IDC) breast tumors and is positively connected with lymph node metastasis, nuclear hormone receptor accumulation, and higher tumor grade (125).

SHP2 deletion inhibits the progression of breast cancer and decreases metastasis (126). Knockout of SHP2 also suppresses ErbB2-induced mammary tumorigenesis (127). In addition, SHP2 knockout attenuates the activation of PI3K/AKT signaling and causes the dephosphorylation and resultant activation of GSK3β in breast cancer cells (128).

In the early phases of triple-negative breast cancer (TNBC), SHP2 plays a crucial mediating role. This is because a shortage in SHP2 activates many SRC family kinases and downstream substrates, which hinder various aspects of tumor cell motility such as invasion, chemotaxis, migration, and velocity (129). A novel substance that suppresses SHP2 and cyclin-dependent kinase 4 (CDK4), downregulates pERK and pRb expression, and boosts immunological function has been shown in recent research to prevent the development of TNBC (130). ETS oncogene 1 (ETS1) activity is decreased in TNBC cell lines when SHP2 is inhibited because it blocks the MAPK signaling cascade, which in turn downregulates IL-8 production (131). In TNBC, SHP2 knockout cells reconstituted with a phosphatase-dead SHP2 mutant are unable to (re-)activate AKT and MAPK signaling upon treatment with BYL719, which makes them sensitive to PI3K inhibition (132). Additionally, drug-resistant cells quickly restart both pathways following PI3K inhibitor monotherapy, whereas combined inhibition of PI3K and SHP2 inhibits proliferation and results in prolonged inactivation of PI3K and MAPK signaling.

Lastly, β-catenin stability might be a further mechanism by which SHP2 promotes TNBC.

In summary, SHP2 plays a vital role in modulating breast cancer development, invasion and metastasis and may be a meaningful therapeutic target in different histological entities of this disease.

### Cervical cancer

In cervical cancer tissues infected with human papillomavirus, SHP2 expression was significantly greater (88.8%) compared to tissues from cervical intraepithelial neoplasia (CIN) (62.5%) and normal cervixes (45%) (133). Furthermore, SHP2 stimulates the initiation and advancement of cervical cancer by preventing IFN-β from being produced, consequently fostering the proliferation of cervical cancer cells (134). Another finding showed that in human cervical cancer cells, SHP2 inhibition has anti-proliferative/anti-migratory effects through the RAS/RAF/ERK and PI3K/AKT pathways (135). Additionally, by triggering autophagy to break down damaged mitochondria and the ubiquitin ligase Parkin to prevent chemotherapy-induced apoptosis in cervical cancer, SHP2 takes part in chemoprotection (136). Through suppressing the activation of PI3K/AKT and NF-κB signaling pathways, the combination of an SHP2 inhibitor and 5-fluorouracil (5-FU) can synergistically induce apoptosis and impede the proliferation of cervical cancer cells (137). In addition, deletion of SHP2 in cervical cancer cells promotes reprogramming of glutamine metabolism (138). When combined, these signaling pathways may allow SHP2 to play an oncogenic function in cervical cancer that can be therapeutically exploited.

### Ovarian cancer

Ovarian cancer cell lines exhibit higher levels of SHP2 expression compared to normal ovarian epithelial cell lines. Additionally, lymph node metastasis, distant metastases, clinical staging, and histological grading were all correlated with SHP2 expression (139). Elevated SHP2 expression in ovarian cancer cell lines has been demonstrated to enhance cell migration, invasion, and proliferation, potentially via the activation of AKT. In contrast, SHP2 inhibition specifically reduced ERK1/2 activity, ovarian cancer cells expressing GAB2, and their ability to proliferate. Furthermore, SHP2 inhibitors and PI3K inhibitors combine to prevent the growth and survival of GAB2-overexpressing ovarian cancer cells both *in vitro* as well as *in vivo* (140). Li et al. (141) noted that the autophagy inhibitor elaiophylin triggers ER-stress and paraptosis by binding directly to SHP2 and thereby triggering toxic hyperactivation of the SHP2/SOS1/MAPK pathway. Then taking advantage of already-elevated MAPK levels in drug-resistant ovarian cancer cells, elaiophylin overcame resistance to chemotherapy in ovarian cancer. Furthermore, very recently, Rossini et al. (142) demonstrated that tyrosine phosphorylation and the association of indoleamine 2,3‐dioxygenase 1 (IDO1) with SHP2 were significantly increased in SKOV-3 cells in response to the IDO1-inhibitor and -stabilizer epacadostat. Meanwhile, in SKOV-3 cells treated with epacadostat the signaling triggered by the transducing molecular complex IDO1-SHP2 accelerates the migratory capacity and the colony-forming ability of SKOV-3 cells, suggesting a pro-tumorigenic phenotype. These findings demonstrate the carcinogenic participation of SHP2 in the formation of ovarian tumors and substantiate the therapeutic promise of targeting this phosphatase in ovarian cancer.

### Prostate cancer

Four distinct prostate cancer cell lines and specimens from 122 prostate cancer patients were examined by Tassidis et al. (143). The findings suggest that enhanced prostate cancer development and progression are linked to the decrease of cytoplasmic SHP2 expression. Conversely, it has been shown that SHP2 is overexpressed in prostate cancer, which is linked to unfavorable clinical outcomes (such as tumor spread and a reduced patient survival time). Increased tumor growth *in vivo*, colony and spheroid formation, and proliferation of prostate cancer cells can result from overexpressing wild-type or oncogenic SHP2 mutations. Furthermore, by weakening the PAR3/PAR6/aPKC polar protein complex as well as promoting EMT, SHP2 can encourage the migration of prostate tumor cells both *in vivo* and *in vitro*, as well as the spread of prostate cancer (144). Targeting SHP2 increases expression of PD-L1 and human leukocyte antigen (HLA) ABC in prostate cancer cells by phosphorylating STAT1, which is relevant to immune-cell interaction and immunotherapy tactics (145).

In conclusion, there is ongoing debate over the involvement of SHP2 in prostate cancer. A more thorough study is needed to identify the precise, and maybe context-dependent, function that SHP2 plays in this disease.

### Melanoma

Common mutations in NRAS and BRAF observed in melanoma activate the RAS/RAF/MAPK and PI3K/AKT signaling pathways (146, 147). Phosphorylated AKT levels exhibit a negative correlation with patient survival rate and rise markedly during the invasion and spread of melanoma (148). Additionally, SHP2 mutations and overexpression were discovered in melanoma patient samples (149). Zhang et al. (150) noted that SHP2 activates the ERK1/2 and AKT signaling pathways to support the survival, motility, and anchoring independent proliferation of melanoma cells. Additionally, they showed that SHP2 suppression slowed the development of xenograft melanoma by enhancing tumor cell death and reducing tumor cell proliferation.

When combined, SHP2 seems to have an oncogenic function in melanoma and presents a viable new target for melanoma combination treatment, which includes tumors with NRAS and BRAF mutations.

### Glioblastoma

The signaling pathways of RTKs, including EGFR, PDGFR, and/or c-MET, are often modified throughout the pathogenesis of glioblastoma multiforme (GBM) (151, 152). In a spontaneous transgenic high-activity HRAS glioma mouse model, inhibition of SHP2 can reduce the development from low-grade astrocytoma to GBM (153). SHP2-dependent pathway inhibition of cellular senescence may be a significant contributing element to establishment of GMB (154). In response to the co-inhibition of EGFR and c-MET, Furcht et al. (155) observed that knocking down SHP2 decreased the proliferative rate of GBM cell lines. SHP2-mediated ERK activity predominated in this context. Furthermore, the co-inhibition of EGFR and c-MET induces cell death, where the SHP2-mediated inhibition of STAT3 plays a crucial role. UBE2D3, a ubiquitin-binding enzyme implicated in the etiology of several malignancies, has the ability to stimulate SHP2 ubiquitination. This, in turn, raises the output of the STAT3 pathway, hence promoting the growth and development of gliomas (156).

In conclusion, selective and careful targeting of SHP2 may have therapeutic implications for the treatment of GBM, given the functional involvement of SHP2 in the onset and progression of the illness.

**Figure 3:**
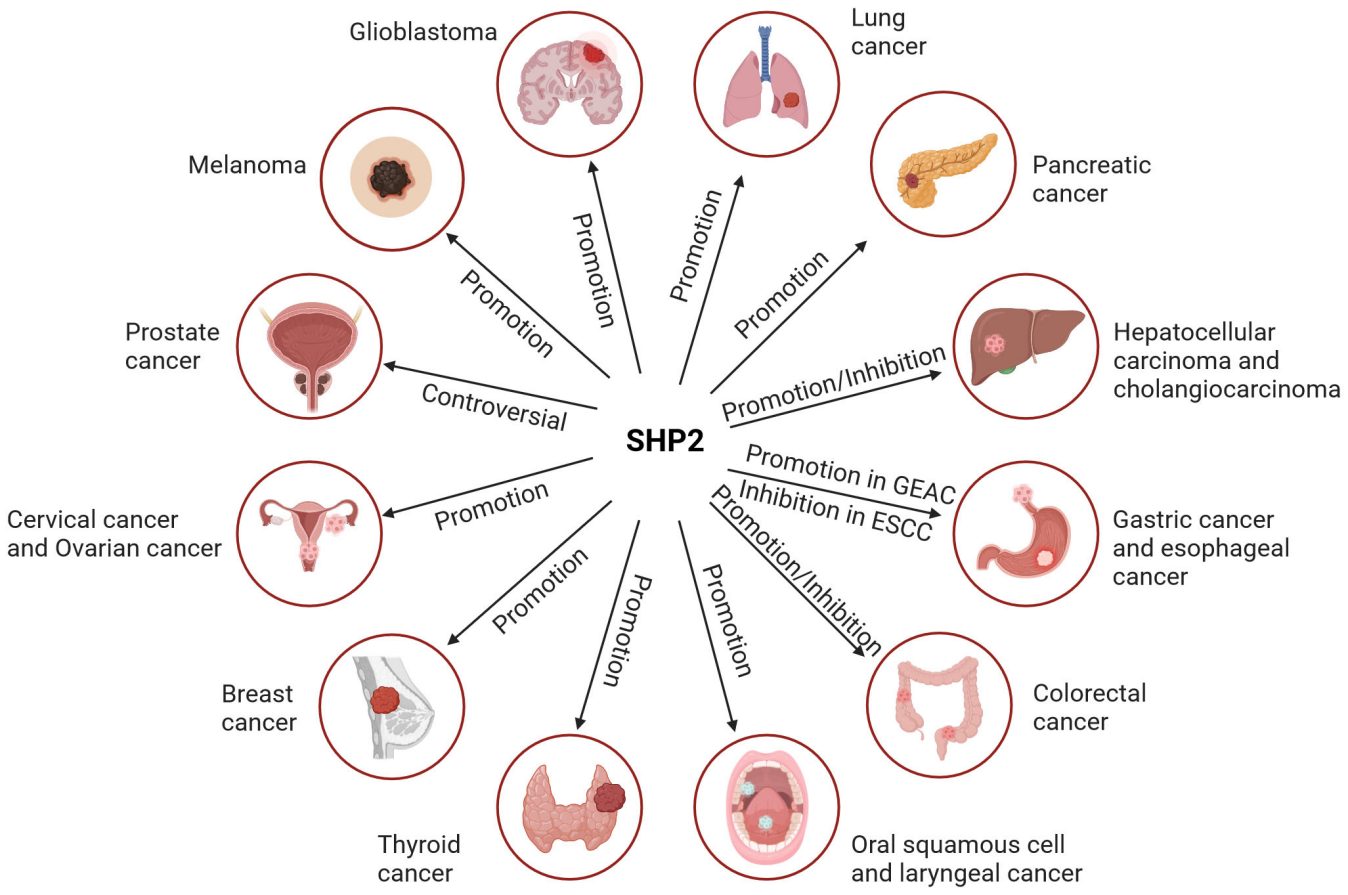
The role of SHP2 in different solid tumor entities. SHP2 primarily serves as a tumor promoter in most solid entities. However, its role in liver and colorectal cancer is dual, encompassing both promoting and suppressing functions. The involvement of SHP2 in prostate cancer remains controversial.

The roles in different solid tumor entities are illustrated in **Figure 3**.

## SHP2 and the cellular tumor microenvironment of solid tumors

Quite some work has been done in the past several years to clarify the significant traits of SHP2 in various cell types within the solid tumor microenvironment. The ubiquitously expressed tyrosine phosphatase regulates signaling pathways and cellular function of all cells present in proximity to and interacting with tumor cells, including immune cells, endothelial cells and fibroblasts, thereby potentially affecting tumor progression.

### Immune cells

#### T-cells

**Figure 4:**
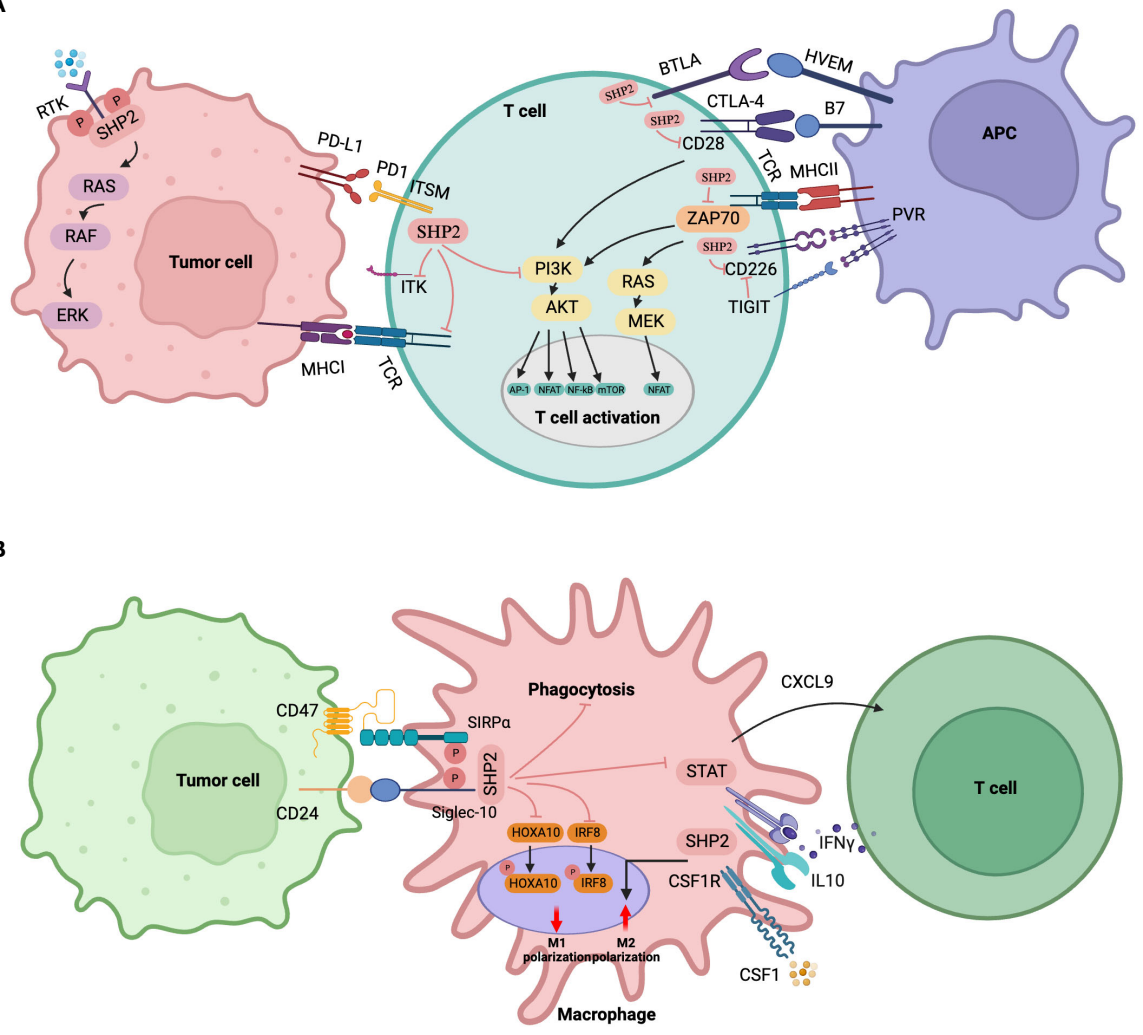
Immune cell-specific roles of SHP2. **(A)** SHP2 in T lymphocytes. SHP2 is recruited by various immunosuppressive receptors like PD-1, CTLA-4, BTLA, TIGIT, etc. The immunosuppressive effects of SHP2 via PD-1 involve (1): direct inhibition of the PI3K-AKT signaling pathway (2); dephosphorylation of ZAP70, indirect inhibition of PI3K-AKT and RAS-ERK pathways (3); dephosphorylation of CD28 to indirectly inhibit the PI3K-AKT pathway (4); For CTLA-4, cytoplasmic SHP2 antagonizes CD28 positive signals, playing a tumor-promoting role (5); PD-1 disrupts CD226 activation through distinct mechanisms when converging with TIGIT (6); SHP2 dephosphorylates ITK downstream of PD-1, inhibiting T cell function. Downstream signaling of SHP2 recruited by BTLA remains unclear. **(B)** SHP2 in myeloid cells/macrophages. SHP2 promotes tumor growth via TAMs through (1): binding to CSF-1-induced signaling complexes, enhancing macrophage proliferation and M2 polarization under CSF-1 stimulation (2); recruiting SIRPα or Siglec-10 receptors to reduce phagocytosis upon CD47 or CD24 stimulation (3); promoting macrophage proliferation via a macrophage/CXCL9-Th1 cell/IFN-γ feedback loop (4); dephosphorylating STAT downstream of IL-10 signaling, inhibiting anti-inflammatory factors, and exacerbating colitis-related colon cancer (5); attenuating GM-CSF-mediated phosphorylation of HOXA10 and IRF8.

SHP2 plays a key role in the regulation of T cell activity by interacting with several immunosuppressive receptors that regulate T cell activation (**Figure 4A**). Numerous immunosuppressive receptors are found in T lymphocytes, including PD-1, B, and T lymphocyte attenuators (BTLA), cytotoxic T lymphocyte-associated antigen-4 (CTLA-4), T cell immunoglobulins, and ITIM domain proteins (TIGIT). These receptors can recruit SHP2 to control T lymphocyte activation through their unique phosphotyrosine motifs (157–160). In cancer immunotherapy, PD-1 is a crucial immune checkpoint target and negative co-stimulatory receptor that is required to prevent T cell activation. Following its interaction with its ligand PD-L1, PD-1 associates with the T cell receptor (TCR), which, in turn, is linked to the phosphatase SHP2. Through the C-terminal tyrosine switch motif (ITSM) present in the immune receptor, PD-1 forms a dimer that interacts with the SH2 domain (N-SH2 and C-SH2) of SHP2. This interaction activates SHP2-mediated immunosuppression, promoting the immune escape of tumor cells (161). Hui et al. (162) showed a preference for SHP2 to dephosphorylate CD28, rather than the TCR, in response to the signaling of PD-1/PD-L1. This suggests that SHP2-mediated inhibition of CD28 signaling is the main mechanism by which PD-1 reduces T cell function. SHP2 is specifically recruited to PD-1 in order to prevent PI3K activation mediated by CD28. This inhibits AKT phosphorylation, subsequently reducing the activation of various transcription factors such as NF-kB, T nuclear factor (NFAT), and activating protein 1 (AP-1), consequently, preventing T cell activity and encouraging T cell malfunction (157). SHP2 may also directly dephosphorylate the co-stimulatory molecule CD226, when it is recruited by PD-1, which limits T cell activation (163). Moreover, it was suggested that SHP2 may suppress T cell activity by dephosphorylating ITK downstream of PD-1 (164). In addition to its impact on CD28 signaling, SHP2 has the ability to stimulate the dephosphorylation of CD3, zeta-chain-associated protein kinase 70 (ZAP70), which in turn inhibits RAS/ERK and PI3K/AKT signaling (165). It is very noteworthy, however, that SHP2 seemed redundant for PD-1 signaling in a series of genetic deletion in *in vivo* tests for tumor immunity and persistent viral infection.

Furthermore, research has shown that BTLA and TIGIT bind SHP2 through the inclusion of an immune receptor tyrosine-based inhibitory motif (ITIM) at the cytoplasmic tail (19, 166).

Additionally, prior studies have demonstrated that the lack of SHP2 in CD4+ T cells stimulates the differentiation of CD4+ T cells into Th1 cells. This results in increased secretion of IFN-γ, activating CD8+ cytotoxic T cells and preventing the onset of colorectal cancer associated with colitis (167). In contrast, additional research discovered that T cells lacking SHP2 had no advantage in halting the development of immunogenic malignancies (158).

SHP2 is most likely to have varied functions in T cells, contingent on the specific environment, signaling pathways, as well as progression stage of T cells. Care should be taken in further analyzing the in-depth effects of SHP2 inhibition on tumor associated T-cell responses.

#### Myeloid cells/macrophages

Angiogenesis, matrix remodeling and metastasis are all significantly influenced by tumor-associated macrophages (TAMs). SHP2 takes part in a number of TAM signaling pathways (**Figure 4B**). In response to colony-stimulating factor 1 (CSF-1), SHP2 binds to the CSF receptor (CSF-1R) complex on the inner membrane of TAMs, thereby activating the RAS/ERK signaling cascade, and in consequence indirectly promoting tumor cell migration, survival, and proliferation (168).

Further, tumor cells can express CD47, which, as a “don’t eat me” signal to the host innate immune system, plays a role in tumor immune evasion. To decrease macrophage phagocytosis and accelerate tumor growth, signal regulatory protein α (SIRPα) may be used to target specific substrates for SHP2 and SHP2 dephosphorylation (169). Li et al. (170) have discovered that the CD47/SIRPα signal triggers substrate deneddylation in colorectal tumor-infiltrating macrophages (TIMs), which lends credence to this perspective. In this instance, deneddylation was necessary for SHP2 to emerge from its own inhibitory state, guarantee activation, and target SIRPα recruitment in order to prevent macrophage phagocytosis.

Barkal et al. (171) demonstrated that the association involving tumor-expressed CD24 and the inhibitory receptor sialic acid-binding Ig-like lectin 10 (Siglec-10) on the surface of TAMs promotes tumor escape. This interaction is initiated by recruiting the immune receptor tyrosine-based inhibitory motif (ITIM) motifs of SHP1 and/or SHP2 to the cytoplasmic tail of Siglec-10. According to a recent study, the Th1-mediated anti-tumor immune microenvironment may be enhanced by the lack of SHP2 in macrophages because it promotes the macrophage/CXCL9 Th1 cells/IFN-γ feedback loop, which in turn inhibits the formation of melanoma (172). Through these pathways, SHP2 loss in macrophages promotes IL10/STAT3 signaling and its reliant anti-inflammatory response, protecting mice against colon cancer invasion caused by colitis (173). Furthermore, the development of B16-F10 melanoma and MC17-51 fibrosarcoma in mice was inhibited by the myeloid-specific deletion of SHP2, and the immunosuppressive potential of myeloid-derived suppressive cells against tumors was decreased (174).

Overall, these results provide strong evidence for a non-autonomous myeloid cell role of SHP2 in tumor progression, suggesting that SHP2 promotes macrophage proliferation and M2-like polarization, encouraging the formation and progression of tumors. Targeting SHP2 in macrophages may be advantageous for M1-like characteristics, phagocytosis, and enhanced antitumor immunity.

#### B-cells

According to a study, SHP2 is a positive regulator of B cell function (175). Following extended IL-6 stimulation, the activation of SHP2 is correlated to the IL-6-induced proliferation of B cells (176). PD-1-mediated activation of SHP2 induces the dephosphorylation of B cell receptor (BCR) proximal signaling molecules, including spleen tyrosine kinase (Syk), phospholipase C gamma 2 (PLCγ2), and ERK1/2 in B cells (177). With a large increase in tumor-infiltrating B lymphocytes, SHP2 depletion suggests that B cells have a considerable anti-tumor impact in NSCLC allogeneic grafts (74). On the other hand, it is unclear from the available data if B cell SHP2 plays a significant role in tumor regulation.

It has been demonstrated that the two ITIM types found in BTLA recruit SHP1 and SHP2, which self-phosphorylate upon binding (166, 178). But SHP1 appears to be used more often than SHP2. Nevertheless, more investigation is required to identify the BTLA signaling mechanism.

#### Other immune cells

Studies have demonstrated that SHP2 may be recruited and activated by certain inhibitory receptors on the surface of natural killer cells (NK) via ITIM motif (179). Excellent cytolytic activity and IFN-γ production in response to tumor target cells are displayed by NK cells devoid of SHP2 (180). However, a recent study discovered that the lack of SHP2 in NK cells had no effect on proliferation and responsiveness of the cells. A decreased rate of SHP2 defective NK cell proliferation and survival was observed when treated alone with high-dose IL-15 or IL-2 (181).

Furthermore, SHP2 also has been ascribed a crucial role in mediating PD-1-regulated cytokine production and NF-κB activation in dendritic cells in an ovarian cancer context (182).

### Endothelial cells

Endothelial cells are structural elements of the vascular system, supplying blood to tissues and becoming ready to react to external harm. They are located on the inside surface of blood arteries. There has been much research done on the function of SHP2 in endothelial processes. SHP2 interacts with VE-cadherin and VEGFR in endothelial cells, which is essential for angiogenesis and the function of the endothelial barrier (183, 184). The context-dependent function of SHP2 as a negative regulator of VEGFR2 signaling and angiogenesis emphasizes the need for more study because VEGF/VEGFR2 regulation is complicated (185). SHP2 modulates the production of pro-angiogenic SRY-Box transcription factor 7 (SOX7) and pathological angiogenesis through apoptosis signal-regulated kinase 1 (ASK1)-c-Jun signaling (186). By downregulating the production of SOX7, which promotes angiogenesis, SHP2 deficiency harms tubular formation, migration, and proliferation of endothelial cells. Tumor development, angiogenesis, and vascular anomalies are all restored in SHP2-deficient animals when SOX7 is re-expressed in SHP2 knockdown cells. Thus, in the tumor vascular system, SHP2 plays a crucial role in altering endothelial cells’ development and survival.

SHP2 promotes proliferative ERK1/2 signaling and suppresses pro-apoptotic STAT3 (187). Systemic SHP2 inhibition increases tumor necrosis, decreases tumor blood vessels and blood perfusion, and promotes tumor vascular system degradation and blood extravasation in animals harboring SHP2-independent tumor cell proliferation of the chosen tumor type.

The current findings highlight the prospect that targeting SHP2 in tumor blood vessel support may be a particularly effective approach and imply that targeting SHP2 may represent a unique strategy for targeting endothelial cells in the tumor vasculature.

### Fibroblasts

Fibroblasts are linked to cancer cells at different phases of the growth of solid tumors, and their structural and functional roles in this process, with all of its intricacies and dual impacts of supporting and restraining tumors, are just now coming to light. With more and more cancer-associated fibroblast (CAF) subtypes being identified, the particular function and contribution of the ubiquitously expressed tyrosine phosphatase SHP2 within each CAF cell type should be carefully elucidated. So far, knowledge for CAFs is very scarce. However, in normal non-tumor-associated fibroblasts, SHP2 appears to play similar roles as e.g. in epithelial cells, being required for full activation of the MAPK/ERK pathway in response to growth factors, FGFs and PDGFs being the most important players for fibroblasts (188). In addition, very recently, Mucciolo et al. (189) demonstrated EGFR-expression and EGFR-dependent activation of a CAF subtype in pancreatic cancer (the myofibroblastic CAFs, or myCAFs), promoting local metastasis. It is very reasonable to hypothesize that SHP2 is involved in signal transduction in this context. To fully decipher the *in vivo* therapeutic benefits of SHP2-inhibition for solid tumors, it is therefore crucial to comprehend the functional role of SHP2 in all different CAF subtypes.

## SHP2 and therapy resistance of solid tumors

Owing to its significance in the signaling pathways discussed above, SHP2 has emerged as a possible target for the management of various solid tumor entities. Historically, mainly due to poor bioavailability and lack of specificity, traditional inhibitors targeting the catalytic site of phosphotyrosine-phosphatases generally displayed unsatisfactory pharmacokinetics and pharmacodynamics. Fortunately, more recently allosteric SHP2 inhibitors have been discovered. The first reported, SHP099, demonstrated high potency, selectivity, solubility and oral bioavailability (190). SHP099 binds between the two SH2 domains and the PTP domain (called the “tunneling” site) to inhibit SHP2 activity by “glueing” it in its inactive state, making SHP099 the first highly selective SHP2 inhibitor. TNO155, RMC-4630, JAB-3068, JAB-3312, ERAS-601, BBP-398, ET0038, and HBI-2376 are just a few of the recently developed, similarly acting allosteric inhibitors that are presently undergoing early stages of clinical trials to assess their tolerability and antitumor effects, either as single agents or in combination therapies. For a concise summary of clinical trials see **Table 1**.

**Table 1:** Overview of currently active clinical trials about SHP2 inhibitors in solid tumors.

NCT Number	Title	Status	Conditions	Interventions	Sponsor	Additional target	Phases
NCT04252339	RLY-1971 in Subjects With Advanced or Metastatic Solid Tumors	Completed	Solid Tumor, Unspecified, Adult	Drug: RLY-1971	Hoffmann-La Roche	–	Phase 1
NCT05525559	SHP2 Inhibitor ET0038 Monotherapy in Patients With Advanced Solid Tumors (FIRST)	Not yet recruiting	Advanced Solid Tumor	Drug: ET0038	Etern BioPharma (Shanghai) Co., Ltd	-	Phase 1
NCT05354843	SHP2 Inhibitor ET0038 Monotherapy in Patients With Advanced Solid Tumors	Recruiting	Advanced Solid Tumor	Drug: ET0038	Etern BioPharma (Shanghai) Co., Ltd	-	Phase 1
NCT05163028	A Dose Escalation Study of SHP2 Inhibitor in Patients With Solid Tumors Harboring KRAS of EGFR Mutations	Recruiting	NSCLC, CRC, PDAC, Solid Tumor, Cancer of Pancreas	Drug: HBI-2376	HUYABIO International, LLC.	-	Phase 1
NCT03518554	A First in Human, Dose Escalation Study of JAB-3068 (SHP2 Inhibitor) in Adult Patients With Advanced Solid Tumors	Recruiting	NSCLC, Head and Neck Cancer, Espphageal Cancer, Other Metastatic Solid Tumors	Drug: JAB-3068	Jacobio Pharmaceuticals Co., Ltd.	-	Phase 1
NCT03565003	A First-in-Human Study of JAB-3068 (SHP2 Inhibitor) in Adult Patients With Advanced Solid Tumors in China	Recruiting	NSCLC, Head and Neck Cancer, Espphageal Cancer, Other Metastatic Solid Tumors	Drug: JAB-3068	Jacobio Pharmaceuticals Co., Ltd.	-	Phase 1/2
NCT05369312	Phase 1 Study of BPI-442096 in Advanced Solid Tumor Patients	Not yet recruiting	Solid Tumor, NSCLC, Pancreatic Cancer, CRC	Drug: BPI-442096	Betta Pharmaceuticals Co., Ltd.	–	Phase 1
NCT05378178	A Phase 1 Study of HS-10381 in Patients With Advanced Solid Tumors	Recruiting	Advanced Solid Tumor	Drug: HS-10381	Jiangsu Hansoh Pharmaceutical Co., Ltd.	-	Phase 1
NCT05621525	Phase 1 Study of the BBP-398 in Patients With Advance Solid tumors	Recruiting	Advanced Solid Tumor, Advanced or Metastatic NSCLC	Drug: BBP-398	LianBio LLC	-	Phase 1
NCT04528836	First-in-Human Study of the SHP2 Inhibitor BBP-398 in Patients With Advanced Solid Tumors	Recruiting	Solid Tumor	Drug: BBP-398	Navire Pharma Inc., a BridgeBio company	-	Phase 1
NCT05480865	SHP2 Inhibitor BBP-398 in Combination With Sotorasib in Patients With Advanced Solid Tumors and a KRAS-G12C Mutaion (Argonaut)	Recruiting	Adult Solid Tumor, Metastatic Solid Tumor, Metastatic NSCLC, NSCLC	Drug: BBP-398, Sotorasib	Navire Pharma Inc., a BridgeBio company, Amgen	KRASG12C	Phase 1
NCT05375084	SHP2 Inhibitor BBP-398 in Combination With Nivolumab in Patients With Advanced Non-Small Cell Lung cancer With a KRAS Mutation	Recruiting	NSCLC, Solid Tumor	Drug: BBP-398 with nivolumab	Navire Pharma Inc., a BridgeBio company, Bristol-Myers Squibb	PD-1	Phase 1
NCT04916236	Combination Therapy of RMC-4630 and LY3214996 in Metastatic KRAS Mutant Cancers (SHERPA)	Recruiting	Pancreatic Cancer, CRC, NSCLC, KRAS Mutation-Related Tumors	Drug: RMC-4630, LY3214996	The Netherlands Cancer Institute, Lustgarten Foundation	ERK1/2	Phase 1
NCT04866134	A Study of ERAS-007 as Monotherapy or in Combination With ERAS-601 in Patients With Advanced or Metastatic Solid Tumors (HERKULES-1)	Recruiting	Advanced or Metastatic Solid Tumors	Drug: ERAS-007, ERAS-601	Erasca, Inc.	ERK1/2	Phase 1/2
NCT04670679	A Dose Escalation/Expansion Study of ERAS-601 in Patients With Advanced or Metastatic Solid Tumors (FLAGSHP-1)	Recruiting	Advanced or Metastatic Solid Tumors	Drug: ERAS-601, Cetuximab, Pembrolizumab	Erasca, Inc.	EGFR, PD-1	Phase 1
NCT03114319	Dose Finding Study of TNO155 in Adult Patients With Advanced Solid Tumors	Recruiting	Advanced EGFR-mutant NSCLC, KRAS G12-mutant NSCLC, ESCC, Head/Neck SCC, Melanoma	Drug: TNO155, TNO155 in combination with EGF816 (Nazartinib)	Novartis Pharmaceuticals	EGFR	Phase 1
NCT04330664	Adagrasib in Combination With TNO155 in Patients With Cancer (KRYSTAL 2)	Active, not recruiting	Advanced Cancer, Metastatic Cancer, Malignant Neoplastic Disease	Drug: MRTX849, TNO155	Mirati Therapeutics Inc. Novartis	KRASG12C	Phase ½
NCT05288205	Phase 1/2a Study of JAB-21822 Plus JAB-3312 in Patients With Advanced Solid Tumors Harboring KRAS p.G12C Mutation	Recruiting	KRAS p.G12C, NSCLC, CRC, PDAC	Drug: JAB-21822, JAB-3312	Jacobio Pharmaceuticals Co., Ltd.	KRASG12C	Phase 1/2
NCT04699188	Study of JDQ443 in Patients With Advanced Solid Tumors Harboring the KRAS G12C Mutation (KontRASt-01)	Recruiting	KRAS G12C Mutant Solid Tumors, NSCLC, CRC, Cancer of Lung	Drug: JDQ443, TNO155, Tislelizumanb	Novartis Pharmaceuticals	KRASG12C, PD-1	Phase 1/2

Importantly, the striking potential of targeting SHP2 encompasses states of therapy resistance of solid tumors in response to chemotherapy and targeted therapies alike.

For instance, it has been shown that SHP2 promotes chemoresistance via cell-autonomous and/or non-autonomous mechanisms. One study observed high expression of SHP2 in chemotherapy-resistant hepatocellular carcinoma (HCC) and patient-derived recurrent HCC samples (98). In another study SHP2 mediated cisplatin resistance by inhibiting apoptosis of lung cancer cells and promoting activation of the RAS/PI3K/AKT/survivin pathway (191). These findings imply that focusing on SHP2 holds promise for overcoming chemotherapy resistance.

A much larger body of evidence has accumulated with respect to inherent and adaptive drug resistance in the context of targeted therapies. In cancer treatment, primary resistance and the emergence of adaptive resistance to small molecule inhibitors are frequent and significant challenges. As a result, creating novel approaches to circumvent and disrupt resistance processes continues to be crucial. For instance, adaptable “drug-resistant” tumor cells can survive if carcinogenic signals are not fully inhibited. These cells can remain in this condition for varying amounts of time before accumulating new genetic alterations linked to acquired resistance and tumor recurrence. SHP2, as was previously indicated, plays a significant role in the signal propagation from virtually all RTKs. It has been discovered that adaptive drug resistance is fueled by the (re)activation of RTK signals in a number of ERK-dependent malignancies, such as RAS mutant tumors, BRAFV600E melanoma, colorectal cancer, thyroid cancer, and TNBC (192, 193). In general, when negative feedback is relieved in response to RAF, MEK, or ERK inhibitors, various RTKs are upregulated and activated (in cell- and context-dependent sets or combinations), which further activates RAS and causes ERK activity to recover, resulting in tumor adaptability, and inhibitor resistance. SHP2 integrates signals from almost all RTKs towards the RAS-RAF-MEK-ERK and PI3K/AKT pathways, and as such, it constitutes a crucial and strategic node in those resistance mediating signaling circuits, even if the relevant collection of RTKs implicated varies depending on the entity and context. It should come as no surprise that several possible applications have been proposed. SHP2 inhibitors, for example, have the ability to stop adaptive resistance to MEK inhibitors (21). The combination of SHP2 and MEK inhibitors has a highly synergistic anti-proliferative impact in KRAS mutant lung cancer and pancreatic cancer (6, 8). Triple-negative cancer, high-grade serous carcinoma, and stomach cancer are examples of hard-to-treat wild-type RAS tumor cells that exhibit adaptive resistance that can be overcome by combining MEK inhibitors with SHP2 inhibitors (107, 194). Combining ERK inhibitors with BRAF inhibitors in several tumor types (195, 196) and BRAF^V600E^ mutated colon cancer (109) exhibits a similar synergism, with SHP2 inhibition as promising additional partner. Furthermore, by delaying the reactivation of RTK-induced MEK/ERK and AKT signaling pathways, SHP099 can be added to sorafenib to avoid adaptive resistance to the drug while treating HCC (99). This was verified by a recent study by Drillon et al. (197), which shows that, in comparison to PF-07284892 (another allosteric SHP2 inhibitor) or each individual targeted treatment regimen, *in vitro* therapy combining PF-07284892 with oncogene matching targeted therapy can enhance the suppression of pERK levels in three human solid tumor cell lines. In mice xenografts of each cell line, oncogene-matched targeted treatment with PF-07284892 produced the greatest tumor reduction in comparison to any one component, which is consistent with pERK suppression.

More recently, the aforementioned findings have been extended to adaptive resistance mechanisms upon inhibition of the PI3K/AKT/mTOR axis. Combining PI3K and SHP2 inhibitors counteracts acquired and intrinsic breast cancer cell resistance to PI3K inhibition mediated by activated receptor tyrosine kinases (132). And in analogy, activation of multiple RTKs upon mTOR inhibition in HCC yields a highly synergistic effect of co-inhibition of both mTOR and SHP2 by triggering apoptosis (100).

With the renewed interest and tremendous effort put into development of RAS-inhibitors during recent years, these drugs, either mutant specific or as pan-RAS strategy are pushing into the clinic. To date, two KRAS^G12C^ inhibitors are FDA-approved and informed by analysis of (pre-)clinical samples and data very similar mechanisms of acquired resistance have already been revealed (198, 199). Based on the mechanisms of resistance described, it is expected that SHP2 inhibitors will have combinatorial value in the context of RAS-inhibition as well.

Lastly, it is realistic to assume and predict the development of resistance to SHP2-inhibition itself, whether monotherapy is used or combination strategies are employed. The reduced ability of the allosteric inhibitors to bind to the phosphatase in its activated open-conformation state is one of its current limitations. Thus, it is thought that, in FGFR-driven cancer for instance, fast FGFR feedback activation following initial suppression of the SHP2 inhibitor pathway promotes the open conformation of SHP2 and results in resistance to SHP2 inhibitors (200). Neel et al. recently presented findings from a CRISPR/Cas9 screen that identified common candidates that contribute to resistance against SHP2 inhibition. In short, functionally the most important drivers were again related to MEK and ERK activity, or RAS stability (201).

Although there is currently a lack of information on evasive mechanisms in response to combination therapy that includes SHP2-inhibition, it is reasonable to assume that epigenetic modifications or mutational processes will again drive reactivation, primarily of the RAS-RAF-MEK-ERK and PI3K-AKT pathways. As was previously noted for KRAS^G12C^ inhibition, an alternative possibility is that the increased inhibitory pressure will cause (some) tumor cells to become independent of these pathways paralleled by evolution of distinct proliferative and survival signals and possibly cellular dedifferentiation and metaplasia (199).

## Discussion and conclusion

Over the past few decades, an in-depth understanding of the molecular structure, functional characteristics, and signaling regulation of SHP2 has been accumulated. Genetic abnormalities of SHP2, including mutations and aberrant expression, are closely associated with leukemia and solid tumors (65). SHP2 plays different roles in various tumors and different tumor microenvironments. Although great progress has been made in the study of SHP2-related mechanisms, the specific processes involved in these mechanisms and context-dependencies need to be further investigated.

SHP2 appears to be (differentially) expressed in various solid tumor tissues such as pancreatic cancer, liver cancer, lung cancer, colorectal cancer, gastric cancer, cervical cancer and breast cancer, and expression levels have been correlated to the occurrence, development and prognosis of tumors. The function of the SHP2 protein is different in distinct contexts. Knockout or inhibition of SHP2 significantly prevented cancer cell proliferation and exerted antitumor activity in NSCLC, PDAC, EGAC, CRC, HCC and others. However, especially in inflammation-driven tumors like some forms of HCC and CRC SHP2 may have intrinsic tumor suppressive effects, and consequently inhibition of the phosphatase would be detrimental in the treatment of these subtypes.

Carefully bearing these limitations in mind, a wide application of SHP2-inhibitors is to envision.

A variety of (allosteric) SHP2 inhibitors have been developed and are currently in investigation in early clinical trials (**Table 1**). Especially in combination approaches with chemotherapy, more importantly with other targeted therapies, and potentially with immunotherapeutic concepts, targeting SHP2 for solid tumor therapy holds promise for an array of tumor entities and indications. However, intrinsic resistance and resistance development will still be a limitation and for SHP2 proving itself as a real “bulls-eye” for targeted therapy of solid tumors patient and tumor stratification in precision medicine contexts will be necessary.

With warranted further research progress, targeting the tyrosine phosphatase SHP2 may establish itself and grow into a powerful dart in the quiver of combination options for the treatment of an array of solid tumors.

## Author contributions

XC: Writing – original draft, Writing – review & editing. SK: Writing – review & editing. PH: Writing – review & editing. AA: Writing – review & editing. TA: Writing – review & editing. JN: Writing – review & editing. JZ: Writing – review & editing. DR: Funding acquisition, Project administration, Writing – review & editing.
